# Commercial Snack Food and Beverage Consumption Prevalence among Children 6–59 Months in West Africa

**DOI:** 10.3390/nu11112715

**Published:** 2019-11-09

**Authors:** Stella Nordhagen, Alissa M. Pries, Romance Dissieka

**Affiliations:** 1 Global Alliance for Improved Nutrition, Rue de Varembé 7, 1202, Geneva, Switzerland; 2 Helen Keller International, Regional Office for Africa, BP 29.898, Dakar, Senegal; 3 Helen Keller International, One Dag Hammarskjold Plaza, Floor 2, New York, NY 10017, USA; apries@hki.org; 4 Helen Keller International, Villa SOPIM 12, le Vallon, J 12, Abidjan, Côte d’Ivoire; rdissieka@hki.org

**Keywords:** complementary feeding, children, snack foods, malnutrition, West Africa

## Abstract

Consumption of commercial snack food and beverage products among infants, young, and school-aged children may have negative effects on child nutritional outcomes, as these foods are typically dense in energy but not in micronutrients. However, there is limited information available about the consumption of such snacks in low-income settings, particularly in Africa. We contribute to filling this gap using data from 11,537 children aged 6–59.9 months from four West African countries (i.e., Burkina Faso, Cote d’Ivoire, Mali, and Niger). We estimated the prevalence of commercial snack food and drink consumption and explored variations within the sample by age group, urban or rural residence, household wealth status, and caregiver educational attainment. The results show that 25.7% of children in Niger, 31.5% in Burkina Faso, 42.9% in Mali, and 45.4% in Cote d’Ivoire ate at least one commercial snack food or beverage in the prior 24 h. Consumption prevalence was significantly higher in urban areas than rural areas, among older children (ages 2–5 y) than those in the complementary feeding period (6–23.9 months), and among children in wealthier households. These relationships were confirmed via logistic regression. Our results confirm the widespread consumption of commercial snack foods and drinks by young children in West Africa, a finding with relevance for nutrition policy and programming.

## 1. Introduction

The growing availability and affordability of unhealthy processed foods globally has coincided with dietary shifts in many parts of the world. Commercial products, including snack foods and sugar-sweetened beverages, are increasingly making up a substantial component of diets [1]. Though plateauing or even falling in many high–income settings, sales and purchases of calories from snack foods and sugar-sweetened beverages have steadily increased across low- and middle-income countries (LMICs) globally in the last 15 years [2]. 

Consumption of commercial snack food and beverage products among infants, young, and school-aged children in low-income settings has been noted [3,4], raising concerns about the effects on child nutritional outcomes, as these foods are typically dense in energy but not in micronutrients. Among infants and young children, overconsumption of these foods could result in micronutrient displacement during a period of heightened nutrient requirements and rapid growth and brain development. Among older pre-school-age children, in addition to potential micronutrient displacement [5], overconsumption has been shown to be associated with increased risk of overweight/obesity due to the excessive energy intakes [6]. Excess consumption of sugary snacks, in particular, could increase the risk of dental caries, which can also lead to worse nutritional status among other outcomes [7,8]. As dietary preferences for sugary or salty foods can be established early in life [6,9,10,11,12], snack consumption at young ages could potentially increase the likelihood of unhealthy dietary patterns (and the risk of overweight/obesity) into childhood and adulthood [6,9,10,11,12].

Despite growing interest in the role that consumption of commercial snack foods might play on both sides of the double burden of malnutrition, there are limited data available on unhealthy diets among children in LMIC contexts where the double burden is growing [13]. There is a particularly stark gap in data for the African continent. Studies have noted the prevalence of commercial snack food consumption among young children in select settings in rural South Africa [14] and urban Senegal and Tanzania [4], but few studies in Africa have captured national-level consumption rates across all age groups of rapid child growth in the first five years of life. In 2018, national surveys across Burkina Faso and Kenya observed that approximately one-third of children 6–59.9 months of age had consumed a sweet snack in the previous 24 hours, with the majority of these sweet snacks commercially processed and packaged [15,16]. While Demographic and Health Surveys (DHS) have captured the prevalence of “sugary food” consumption in many countries across Africa [17], these data are not consistently collected across survey rounds and only among children 6–23 months of age. In addition, this measure imperfectly captures only one type of snack food while missing measurements of savory snack foods and sugar-sweetened beverages entirely.

This paper aimed to help fill this data gap by examining commercial snack food consumption among children ages 6 months to 5 years in four West African countries: Mali, Cote d’Ivoire, Niger, and Burkina Faso. In the following sections, we present the prevalence of commercial snack food consumption for sugary and savory snacks and for sugary drinks. We then explore variations within the sample by age group, urban or rural residence, and household wealth status. West Africa is an important region in which to examine the question of commercial snack food consumption given the persistent undernutrition alongside growing urbanization and reliance on markets [18] and the increasing prevalence of the double burden of malnutrition [19,20] and diet-related Type II diseases [21]. Building understanding of the presence of unhealthy commercial food products within the diets of children will be increasingly important for stakeholders aiming to develop policies related to food systems regulation and those striving to improve diet quality and protect healthy diets among the youngest population. 

## 2. Material and Methods

This study made opportunistic use of a series of four cross-sectional population surveys intended to measure vaccination and vitamin A supplementation (VAS) coverage. During these four surveys, data were also collected on consumption of commercial snack foods among children aged 6–59.9 months in Burkina Faso, Cote d’Ivoire, Mali, and Niger. The status of child malnutrition and complementary feeding practices in each of these countries is summarized in Table 1.

The surveys followed two versions of the World Health Organization’s (WHO) recommended vaccination coverage survey methodology [26], using two-stage cluster random sampling. In each, a set of strata was determined based on local geographies and health systems, and a number of clusters per stratum was randomly and independently chosen, without replacement, from census Zones de Dénombrement (ZD). Next, all households in the ZD with children aged 6–59.9 months were listed, using a door-to-door census, and approximately 10 households per ZD [26] were chosen using a random number generator. Additional replacement households were also randomly chosen in case those initially selected declined to participate. In approximately 30% of ZDs in Cote d’Ivoire, fewer than 9 or more than 11 households were included due to the fact of either enumerator error or difficulty finding selected households. This variation was accounted for in the weighting of estimates. All sampling used the most recent national census data (Cote d’Ivoire: 2014; Niger: 2012; Burkina Faso: 2006; Mali: 2009). Within each household, data were collected on all children aged 6–59.9 months.

In Cote d’Ivoire, the survey covered 65 (of 82 total) health districts, which were evenly distributed across the country and covering all its major zones, and divided into three strata with up to 96 clusters per stratum (about half in urban areas and half in rural areas), depending on stratum population. In Niger, sampling covered 43 (out of 72 total) health districts divided in two strata and uniformly distributed throughout the accessible areas of the country, taking into consideration the security situation at the time of data collection. Seventy-seven clusters per strata (154 total) were randomly selected, divided across rural and urban areas in each stratum proportionate to the urban–rural population. In Burkina Faso, the survey was conducted in 14 (of 70 total) health districts divided into three strata (the health regions Centre-Ouest, Centre-Sud, and Plateau-Central). In each stratum, 77 clusters were randomly selected (231 total), distributed across rural and urban areas according to census data. In Mali, sampling covered five of the country’s ten regions, divided in two strata. The regions covered (Kayes, Segou, Bamako, Koulikoro, and Sikasso regions) are generally representative of southern Mali, where the majority of the country’s population lives; northern Mali was excluded due to the security concerns. In each stratum, 77 clusters were randomly selected from the census list of 154 clusters, proportionally distributed across urban and rural areas.

Data were collected via face-to-face structured interviews with caregivers at the household level in local languages by trained interviewers. No personally identifying details on respondents were collected. Data were collected electronically using tablets and the Android application Open Data Kit and uploaded to a secure, password-protected cloud-based server. All subjects gave their informed consent for inclusion before responding to the survey. The study was conducted in accordance with the Declaration of Helsinki, and ethical approval for the research in each country was granted by the relevant national research ethics committees (Cote d’Ivoire Comité National d’Ethique des Sciences de la Vie et de la Santé, #223-2018; Mali Comité d’Ethique de l’Institut National de Recherche en Santé Publique, #14-2018; Niger Comité National d’Ethique pour la Recherche en Santé, #017/2019; Burkina Faso Comité d’Ethique pour la Recherche en Santé, #2019-5-065).

The questionnaire included measures of basic sociodemographic characteristics, receipt of health services (including children’s VAS and vaccination status), health-seeking behaviors, and consumption of commercial snack foods during the day prior to the interview. These included three categories of commercial products: (1) sugary snack food products, (2) savory snack food products, and (3) sugar-sweetened beverages. The questions on commercial snack foods were as follows, with probes of context-specific product examples provided:

“Yesterday, during the day or night, did [Child’s name] consume:

- Any commercially produced/packaged sugary foods such as chocolates, sweets, candies, pastries, cakes, or sweet biscuits?

- Any commercially produced/packaged soft drinks, soda, or fizzy drinks?

- Any commercially produced/packaged savory snacks such as fried chips, crisps, or salted biscuits?”

The questions were identical across countries, although examples were adapted to align with local product availability and preferences. One hundred percent fruit juice was not included in the beverage category but juice drinks were included. These questions were adapted from previous studies in Senegal and Tanzania [27,28], and were used in national level questionnaires in Kenya and Burkina Faso [15,16]. Data were collected in November 2018 in Cote d’Ivoire, May 2019 in Niger, and July 2019 in Burkina Faso and Mali.

Analysis was undertaken using Stata SE15 [29]. Data were cleaned for aberrant or implausible values, labelled, and analyzed using descriptive statistics and two-sided Pearson’s chi-squared tests to compare proportions across groups based on variables found to be significantly associated with snack consumption in prior studies [4] including: wealth status, caregiver educational attainment, and child age. Specifically, a wealth index was created using principal components analysis. This was based on questions related to housing conditions, sanitation access, fuel type, and various assets; the assets used varied across the countries, depending on relevance in the local context. These responses were re-coded into binary variables and those with little variation (> 95% or < 5% of respondents) were excluded from the analysis. Improved water and toilet status were defined based on the standards of the Joint Monitoring Program for Water Supply and Sanitation of UNICEF and WHO (2012). Factors were retained based on inspection of a scree plot and an eigenvalue cut-off of 1.0 and then rotated to create a continuous index which was then broken into quartiles. Given the wide variations in living conditions and wealth between urban and rural areas, the index was created separately for urban and rural households. Educational attainment focused on completion of primary school as defined under the education system in each country. Specific age groups (6–23.9 months, 12–23.9 months, and 2–5 years) were also explored, with groupings of young children in line with those used in WHO infant and young child feeding measures [30]. 

Multivariate logistic regressions were used to predict consumption of each of the three snack food categories based on a set of covariates: the child’s age in months, the wealth index, and binary indicators of whether the child was female, whether the household was in a rural area, and whether the respondent had completed primary school. Results were adjusted for clustering at the district level, and the model was selected among different alternative specifications for wealth (and its interaction with urban residence) based on the pseudo-*R*^2^ value. Probability weights were created for each household and used to weight the estimates following WHO (2015).

## 3. Results

The total sample size was 11,537 children aged 6–59.9 months: 3538 in Cote d’Ivoire, 2212 in Mali, 2556 in Niger, and 3231 in Burkina Faso. A summary of the basic demographic information on respondents and their households is given in Table 2. Respondents were typically women in their early 30s who had not completed primary school. Respondents were generally the mother of the child, though other adult women (grandmother, aunt) were also common (particularly in Cote d’Ivoire, where 33% were other adult caregivers). Most households had access to improved water facilities but only a minority had access to improved toilets. About half of households in the Cote d’Ivoire sample were in urban areas, whereas only about 10% were in the other samples. Children were, on average, 2–2.5 years old and split evenly between boys and girls. 

Results for commercial snack food consumption prevalence are shown in Table 3, presented by urban and rural areas and by the complementary feeding period (ages 6–23.9 months) and pre–school age children (ages 2–5 years). For ease of interpretation and due to the similarities between foods as opposed to drinks, we grouped together sugary and savory snacks; across nearly all settings, sugary snack consumption was much higher than savory snack consumption. Overall, 25.7% of children in Niger, 31.5% in Burkina Faso, 42.9% in Mali, and 45.4% in Cote d’Ivoire (across all age groups) had eaten at least one of the three types of commercially produced snack foods in the 24 hours prior to the survey. Most of these children consumed snack foods or drinks from only one of the three categories, but 7.5% of the sample in Mali and 10.7% of the sample in Cote d’Ivoire had consumed foods from all three categories. Niger had the lowest levels of consumption prevalence among all the countries examined, whereas Cote d’Ivoire had the highest. As Table 3 shows, there was considerable and systematic variation by age and residence zone. Consumption prevalence was universally significantly higher (*p* < 0.05) in urban versus rural areas and among older (24–59 months) versus younger children (6–23 months). Sugary snacks were the most commonly consumed category across all ages and areas. There were very few significant differences in consumption prevalence between girls and boys for any snack food category or age group (results not shown).

**Figure 1:**
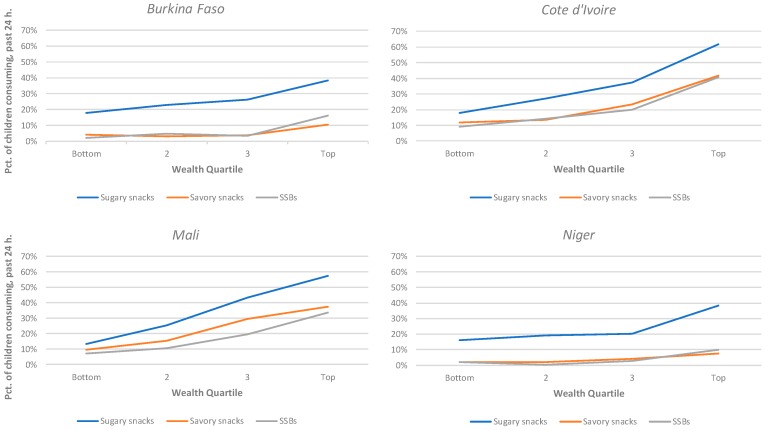
Percentage of children consuming snacks and sugar-sweetened beverages (SSBs) in past 24 h by wealth quartile.

Table 4 and Figure 1 present the association between snack food consumption prevalence and household wealth quartile. Though there were some variations in the exact trend, the overall relationship was similar across contexts. In most countries and in urban and rural areas, consumption prevalence was higher among children in wealthier households, though this difference was not always significant, likely due in part to the limited power once sample sizes were disaggregated by both age groups and wealth groups. This trend was seen for all three snack categories. The exception to this was in rural Cote d’Ivoire, where the prevalence of consumption initially rose slightly with wealth but was lower among the top wealth quartile (data not shown). In Niger and Burkina Faso, there were limited differences across the bottom four wealth quartiles but significantly higher rates within the top quartile, whereas Mali and Cote d’Ivoire showed a steadier upward trend across wealth quartiles. Prevalence rates for children in the poorest 25% of urban households were similar to those observed in the richest 25% of households in rural areas in Burkina Faso and Cote d’Ivoire; in Mali and Niger, however, the levels seen among children in the wealthiest rural households generally remained below the levels for all households in urban areas.

We also examined associations between a child’s commercial snack consumption prevalence and caregiver’s completion of primary school (as opposed to non-completion; results not shown). Results indicated that consumption prevalence trended higher among children of more educated mothers, with that difference being significant in most cases. Higher levels of education were not considered due to the small sample sizes entailed (e.g., 7.9–18.7% of respondents by country had completed secondary school or higher, with those completing concentrated in urban areas). 

Multivariate logistic regressions predicting consumption prevalence of commercial snack foods were used to clarify these bivariate relationships. Table 5 presents the regression results, displayed as odds ratios. Across all countries, child age was positively and significantly associated with consumption prevalence, with older children being more likely to consume commercial snacks than younger children, but there was no significant relationship with child sex. The respondent’s completion of primary school was significantly and positively associated with higher odds of snack food consumption across most categories relative to respondents who had not completed primary school. As a robustness test, this was repeated including only observations for which the respondent was the child’s mother, making this variable an indicator for maternal education. The results (not shown) were qualitatively similar. Probability of consumption was significantly higher for those in the top quartile of wealth for all snack/drink categories in Burkina Faso and Cote d’Ivoire and for all except savory snacks in Niger and Mali. Multicollinearity of covariates was explored by assessing uncentered variance inflation factors; no VIF was above 4.0, indicating minimal multicollinearity.

## 4. Discussion

Our results from surveys spanning urban and rural areas of four countries confirm that consumption of commercial snack foods and drinks among young children in West Africa is prevalent, ranging from 25.7% eating at least one type of snack food in the prior 24 hours in Niger to 45.4% in Cote d’Ivoire. Sugary snacks were the most commonly consumed category, far ahead of savory snacks or SSBs in all contexts. Prevalence of consumption was observed to trend higher among older children, in urban areas, among children whose mothers had completed primary school (as compared to less or no education), and among the wealthiest households. This analysis provides an understanding of how common consumption of commercially processed, packaged foods, and beverages is within diets of young and pre-school age children in the West Africa region. 

Despite limited data within Africa, and even more limited within the West Africa region specifically, the snack consumption prevalence rates presented here for children 6–23 months are comparable to rates noted in other prior studies. We report rates ranging from 21.4–38.4% which is similar to the 42% savory snack consumption reported among 6–12 month-olds in rural KwaZulu-Natal, South Africa [14] and comparable to the 23% and 59% commercial snack food consumption among 6–23 month-olds reported in 2007 for Dar es Salaam, Tanzania, and in Dakar, Senegal, respectively [27,28]. Similarly, Huffman et al. [17] used DHS data from 2006–2011 to examine consumption of sugary foods among children ages 6–23.9 months, showing that between 21%–38% of children in six West African countries had consumed such foods in the prior 24 hours. Consumption rates in seven East African countries were generally lower, 11%–25%, with the exception of Swaziland (40%). Consumption of sugary snacks was higher than fortified infant cereals or eggs and vitamin A–rich fruits. That analysis did not examine commercial snacks, specifically, nor did it consider non–sweet foods. More recent nationally representative surveys conducted by Performance Monitoring and Accountability (PMA) 2020 observed that 11%, 38%, and 11% of Kenyan children 6–23 months had consumed savory snacks, sweet snacks, and sugar-sweetened beverages, and consumption prevalence rates of 6%, 25%, and 12% among 6–23 month-olds were observed in Burkina Faso, respectively [15,16]. The present research provides additional data on this topic beyond urban areas and in a broader range of countries, confirming that consumption of commercial snack foods is widely prevalent during the complementary feeding period and among ages 2–5 years across countries. That consumption prevalence rates were fairly high even for rural areas of some of Africa’s least-developed countries is striking.

Commercially produced snack food products are often micronutrient-poor and high in salt/sugar [31,32,33] and trans fats [34] making them inappropriate for infant and young child feeding [34]. Early in life, overconsumption of foods high in energy density but low in nutrient density could displace consumption of other nutritious foods, including breastmilk [35], thereby potentially leading to inadequate intakes of micronutrients. Previous studies [36,37,38,39,40] have shown an association between consumption of snack foods/beverages and displaced consumption of other nutrient-rich foods and/or reduced nutrient intakes in high-income settings. Micronutrient-density of complementary feeding diets among young children in low- and middle-income settings is often limited, and so the risk of micronutrient displacement and potentially inadequate intake is even higher in these contexts. Though little research has been done on the relationship between snack food consumption and dietary adequacy during the first two years of life in such contexts [13], one recent study in Kathmandu Valley, Nepal, of 12–23 month-olds observed lower nutrient intakes and mean probability of dietary adequacy among the highest consumers of unhealthy snack foods and beverages [41]. Given the low proportion of children under 2 years achieving the minimum recommendation for dietary diversity in the four countries included in this present study, there is a plausible and substantial risk of micronutrient dilution in the diet, which could contribute to dietary inadequacy, from consumption of commercial snack foods and beverages. 

High consumption of unhealthy snack foods and beverages has also been linked to increased risk of overweight/obesity through several mechanisms. In addition to contributing to weight gain and overweight/obesity through high energy and fat intakes [42,43], several other mechanisms serve as pathways to overnutrition. Evidence shows that satiety levels when consuming sugar-sweetened beverages are lower than when consuming non-sweetened beverages [44,45], thereby resulting in excessive caloric intakes. Additionally, dietary preferences for sugary or salty foods are established early in life [6,9,10,11,12], potentially forming unhealthy dietary patterns that can continue into childhood and adulthood and increase the risk of overweight/obesity [6,9,10,11,12]. Globally, 40 million children are overweight/obese, with the majority of these children living in low- and middle-income countries, where the rate of increase of overnutrition is increasing most rapidly [46].

The increase in children’s snack food consumption with wealth aligns with results found by Huffman et al. [17] in secondary data analysis among several countries in West Africa (2017). Given the low levels of economic development and widespread poverty in these countries, this is not surprising: the poorest families may lack the income to buy even cheap snack foods. However, a recent study among young children in urban Nepal noted a negative relationship between wealth status and contribution of commercial snack foods to total energy intake, with the poorest children having greater odds of having diets dominated by these products [41]. Such differing results may be explained by context or by different measurements of consumption. While a greater proportion of wealthy children may consume such products, poor families may rely more heavily on cheap processed foods for calories, given the higher cost of healthy foods [22]. It should also be noted that the wealth index used may not be a strong proxy for wealth in rural areas, as several of the constituents (e.g., electricity, water, toilet type) are related more to village-level infrastructure and location than to individual variations across households within a village. Higher rates of consumption prevalence in urban areas are also not unexpected and may relate to several factors: greater prevalence of snack foods in the food environment, more exposure to advertising and promotion of such foods, less time for food preparation due to the caregivers’ employment outside the home, and different cultural norms around children’s diets. 

The positive association between caregiver completion of primary education and consumption is a surprising finding at odds with some prior research [4]. There are a number of potential explanations, including that commercial snack foods are “aspirational”, considered a sign of a modern, upper-class diet; that higher educated caregivers are more exposed to food advertising and promotion due to the purchasing or entertainment-viewing habits; or that more educated caregivers may have less time to cook and, thus, be more reliant on purchased snacks. All of these could also be related to the increasing levels of consumption with wealth, as wealth and education are highly correlated-though the association persisted in the regression analysis after wealth and urban residence were controlled for. The association could also be an artifact of better-educated respondents having a clearer understanding of the question and, thus, reporting more accurately. In addition, the relationship between educational attainment and commercial snack consumption may be differential depending on the level of education attained; as noted, due to the limited power, we were not able to explore the relationship among secondary or university level educational attainment, which may have a different directionality for the relationship. Such possibilities should be explored in more depth in future research in the West African region. 

This analysis has several limitations. First, the data presented here are admittedly basic: it gives no indication of the quantity of snack foods or beverages consumed, the specific nutritional value of products consumed, or the wider diet within which these foods are consumed. It also excludes homemade snack foods that may also be of limited nutritional value, and the data cover only one 24 h period which may not be representative of the normal diet. Despite this, prevalence data on consumption trends are important to observe shifts in overall diet patterns and trends across regions and countries. Given the limited data on this topic and for this age group (particularly in West Africa), we feel these results provide a useful overview that can spur additional research to provide more detailed dietary information in these populations. Second, only certain regions of each country were covered. Particularly in Mali and Niger, certain regions of the country were not covered due to the presence of security issues; as these tended to be more remote and poorer, it is likely that they would have had lower consumption prevalence. The different coverage areas across countries may have contributed to the variation seen across countries. Third, although standard phrasings and locally specific, comparable examples of “commercially produced/packaged” foods were used, the phrase may not have been understood identically across contexts. Finally, data were collected at different times of year across the different countries; it is unknown how and whether seasonality impacts commercial snack food consumption. Despite these limitations, the large sample size and random population-based sampling provide a useful overview of children’s commercial snack food consumption in West Africa which can be the basis for more in-depth future research.

Indeed, we see considerable potential for expanding upon this research in the future. Additional survey work can seek to obtain more detailed data on the quantities of snack foods consumed, their nutrient content, and how such foods fit within the rest of the diet. There is also a need to devise and validate better indicators for snack food consumption among young children that can better differentiate between healthy and unhealthy snacks including homemade options. In-depth qualitative research could probe caregivers’ motivations for feeding such foods to young children, whereas comparisons with data on child growth or micronutrient status could help unpack the role that commercial snack food consumption plays in determining nutritional status among young children in LMICs. Mixed-methods work could try to identify whether there are key ages in early childhood when food preferences are formed (and when eating sugary or savory snacks could have a particular influence on these preferences). Finally, trials could evaluate strategies to limit snack food consumption among young children in LMICs and/or guide food purchase and consumption decisions towards more nutritious foods. The recently revised Demographic and Health Survey (DHS-8) questionnaire offers an opportunity to expand research on snack food consumption by young children in LMICs, as it now includes three questions on snack and SSB consumption. 

With more information on the impact of snack food consumption on young children’s diets and nutritional status, policymakers can be better positioned to identify the steps needed to reduce consumption. Such approaches could include reformulation or the creation of standards for such products; taxes; bans on advertising and sales of such products in public places; limitations on marketing related to children (such as the images of babies commonly placed on biscuit packaging); or nutrition education campaigns. However, dealing with the more fundamental problem of low-quality diets among young children requires addressing the basic barriers to the consumption of nutritious foods: limited availability and, particularly, affordability of such foods for many households [47].

## 5. Conclusions

This paper used data from 11,854 children aged 6–59.9 months from four West African countries to examine the prevalence of commercial snack food consumption during this critical growth period and how it varied with child age, household wealth, urban/rural residence, and caregiver’s educational attainment. The results showed fairly high levels of consumption prevalence (25.7–45.4%) which was systematically higher in urban areas, among wealthier households, and for children aged 2–5 years (as opposed to 6–23.9 months). These results have potential implications for both sides of the nutrition “double burden” and underline the importance of greater focus on how to steer parents to healthier food choices, which will likely require a combination of policy nudges and broader attempts to reshape food systems to improve the availability, accessibility, and affordability of nutritious foods.

## Figures and Tables

**Table 1:** Child malnutrition in four target countries.

	Burkina Faso	Cote d’Ivoire	Mali	Niger
Stunting among children 6–59 months	Rural: 37.3% Urban: 21.3%	Rural: 34.9% Urban: 20.5%	Rural: 41.9% Urban: 23.2%	Rural: 45.9% Urban: 29.6%
Anemia among children 6–59 months	Rural: 89.9% Urban: 77.6%	Rural: 79.3% Urban: 67.2%	Rural: 84.9% Urban: 67.5%	Rural: 69.8% Urban: 73.9%
Minimum dietary diversity among children 6–23 months	Rural: 3.9% Urban: 11.5%	Rural: 5.8% Urban: 20.0%	Rural: 31.3% Urban: 49.7%	Rural: 6.4% Urban: 30.4%

Sources: Demographic and health surveys [22,23,24,25].

**Table 2:** Summary of respondents’ characteristics.

	Burkina Faso	Cote d’Ivoire	Mali	Niger
Caregiver age (y) *	33.0 (32.2–33.8)	33.2 (32.3–34.1)	31.7 (30.8–32.5)	31.3 (30.5–32.1)
Caregiver sex (% female)	86.3% (2801)	83.4% (2918)	84.4% (1885)	91.3% (2600)
Child age (m) *	31.8 (31.2–32.3)	32.6 (31.9–33.3)	31.0 (30.4–31.6)	30 (29.5–31.1)
Aged 6–23 (m)	36.9% (1172)	35.1% (1263)	32.3% (720)	26.3% (1030)
Aged 2–5 (y)	63.1% (2059)	48.9% (2275)	67.7% (1492)	73.7% (1805)
Child sex (% female)	48.0% (1562)	48.6% (1707)	49.2% (1086)	50.9% (1416)
Urban residence (%)	10.3% (666)	52.8% (1738)	26.7% (1509)	7.9% (347)
Use of improved water source	90.2% (2947)	83.9% (2946)	74.3% (1679)	61.5% (1769)
Improved toilet access	23.1% (860)	27.6% (918)	35.0% (850)	9.6% (343)
Caregiver completed primary school	25.3% (935)	39.5% (1315)	36.5% (836)	17.9% (558)
*n*	3231	3538	2212	2556

* Weighted estimates provided; mean (95% confidence interval) or percentage (*n*).

**Table 3:** Percentage of children consuming commercially packaged snacks in prior 24 h.

**Ages 6–23.9 months**																
	**Burkina Faso**				**Cote d’Ivoire**				**Mali**				**Niger**			
	Rural	Urban	Total	*p*	Rural	Urban	Total	*p*	Rural	Urban	Total	*p*	Rural	Urban	Total	*p*
Sugary drinks	2.8% (27)	17.9% (43)	4.3% (70)	0.000	9.1% (51)	21.7% (102)	15.4% (153)	0.000	8.9% (42)	28.4% (73)	14.6% (115)	0.000	1.6% (7)	8.9% (6)	2.3% (13)	0.001
Savory or sugary snacks	19.3% (182)	38.7% (89)	21.3% (273)	0.000	26.1% (149)	42.0% (218)	34.0% (367)	0.000	26.7% (125)	61.8% (142)	36.7% (267)	0.000	17.9% (116)	48.0% (53)	20.7% (169)	0.000
Any	20.3% (191)	45.3% (106)	22.8% (299)	0.000	27.6% (159)	45.9% (236)	36.7% (395)	0.000	27.9% (132)	64.2% (149)	38.4% (281)	0.000	18.6% (118)	49.0% (54)	21.4% (172)	0.000
n	937	235	1172		670	593	1263		477	250	727		687	108	795	
**Ages 2–5 years**																
	**Burkina Faso**				**Cote d’Ivoire**				**Mali**					**Niger**		
	Rural	Urban	Total	*p*	Rural	Urban	Total	*p*	Rural	Urban	Total	*p*	Rural	Urban	Total	*p*
Sugary drinks	5.2% (84)	24.9% (107)	7.3% (191)	0.000	13.3% (125)	36.2% (337)	25.8% (462)	0.000	12.6% (126)	36.3% (170)	18.5% (296)	0.000	3.3% (45)	19.9% (42)	4.6% (87)	0.000
Savory or sugary snacks	27.1% (429)	54.1% (235)	29.9% (667)	0.000	30.0% (282)	61.3% (598)	47.0% (880)	0.000	34.3% (328)	72.3% (303)	43.7% (631)	0.000	27.2% (402)	64.9% (140)	30.2% (542)	0.000
Any	27.8% (440)	60.1% (259)	31.2% (702)	0.000	32.5% (308)	64.9% (640)	50.1% (948)	0.000	36.2% (347)	76.5% (324)	46.3% (671)	0.000	27.2% (406)	65.6% (141)	30.4% (547)	0.000
*n*	1607	452	2059		1138	1175	2313		1032	460	1492		1605	221	1826	

Notes: *p*-values are for Pearson’s Chi-Squared test for a significant difference between urban and rural rates for the given age category and country, corrected for weighting using the second-order Rao and Scott correction. Percentages and *n* do not align, because percentages are weighted whereas *n* are raw numbers.

**Table 4:** Percentage of children consuming commercial snack foods in past 24 h by wealth quartile in rural and urban areas.

**RURAL AREAS**												
**Ages 6–23.9 months**												
	**Burkina Faso**			**Cote d’Ivoire**			**Mali**			**Niger**		
	Bottom	Top	*p*	Bottom	Top	*p*	Bottom	Top	*p*	Bottom	Top	*p*
Sugary drinks	0.5% (1)	3.8% (10)	0.032	8.4% (51)	7.7% (102)	0.872	5.5% (7)	13.5% (15)	0.0319	1.8% (1)	0.5% (2)	0.259
Savory or sugary snacks	9.9% (17)	22.6% (60)	0.004	24.5% (149)	20.7% (218)	0.526	14.6% (17)	36.0% (41)	0.000	8.0% (12)	26.6% (45)	0.000
Any	9.9% (17)	24.6% (65)	0.001	25.8% (159)	24.0% (236)	0.782	16.8% (20)	38.3% (44)	0.001	8.0% (12)	26.6% (45)	0.000
*n*	171	262		145	163		118	119		146	182	
**Ages 2–5 years**												
Sugary drinks	1.2% (4)	7.6% (31)	0.000	12.3% (25)	20.0% (337)	0.202	8.7% (22)	21.7% (53)	0.002	1.0% (3)	8.0% (25)	0.001
Savory or sugary snacks	12.9% (42)	39.3% (160)	0.000	26.8% (282)	35.7% (598)	0.119	16.6% (39)	53.8% (128)	0.000	13.5% (46)	42.5% (145)	0.000
Any	12.9% (42)	45.3% (163)	0.000	29.1% (308)	38.0% (640)	0.104	18.7% (44)	57.8% (137)	0.000	13.8% (47)	42.2% (145)	0.000
*n*	318	404		282	268		250	259		351	391	
**URBAN AREAS**												
**Ages 6–23.9 months**												
	**Burkina Faso**			**Cote d’Ivoire**			**Mali**				**Niger**	
	Bottom	Top	*p*	Bottom	Top	*p*	Bottom	Top	*p*	Bottom	Top	*p*
Sugary drinks	2.8% (2)	44.8% (24)	0.0000	11.5% (10)	32.6% (52)	0.005	20.6% (17)	25.5% (17)	0.5851	6.4% (1)	4.7% (1)	0.8327
Savory or sugary snacks	39.6% (23)	58.8% (31)	0.0703	19.4% (28)	55.5% (92)	0.000	43.7% (29)	71.8% (40)	0.0104	37.2% (11)	67.3% (16)	0.1081
Any	41.3% (24)	73.6% (40)	0.0002	24.1% (32)	58.1% (96)	0.000	46.3% (31)	68.9% (40)	0.0698	37.2% (11)	67.3% (16)	0.1081
*n*	56	55		159	174		62	60		28	23	
**Ages 2–5 years**												
Sugary drinks	9.6% (11)	41.1% (48)	0.0001	17.4% (36)	48.8% (144)	0.000	26.6% (34)	40.2% (47)	0.0303	13.3% (6)	23.8% (16)	0.3463
Savory or sugary snacks	41.3% (44)	69.2% (82)	0.0009	33.7% (73)	74.8% (221)	0.000	60.2% (59)	72.5% (84)	0.1395	59.7% (29)	80.1% (49)	0.1661
Any	41.9% (45)	78.4% (93)	0.0000	35.1% (78)	80.3% (238)	0.000	64.4% (65)	79.7% (94)	0.0488	59.2% (29)	80.1% (49)	0.1551

Notes: *p*-Values are for Pearson’s Chi2 test for a significant difference between rates in the bottom and top quartiles for the given age category and country, corrected for weighting using the second-order Rao and Scott correction. Percentages and *n* do not align, because percentages are weighted whereas *n* are raw numbers.

**Table 5:** Results of multivariate logistic regressions predicting children’s snack consumption, past 24 h.

**Cote d’Ivoire**						
	**Savory or sugary snacks**				**SSBs**	
	**Odds ratio**	**95% CI**	***p***	**Odds ratio**	**95% CI**	***p***
Female child	1.044	0.873–1.250	0.634	1.043	0.849–1.281	0.688
Child age (m)	1.019	1.013–1.025	0.000	1.022	1.015–1.029	0.000
Respondent completed primary education	1.500	1.206–1.866	0.000	1.422	1.057–1.913	0.020
Households in top wealth quartile	3.072	2.350–4.014	0.000	2.666	1.874–3.794	0.000
Urban area	1.751	1.249–2.455	0.001	1.928	1.287–2.889	0.002
Constant (baseline odds)	0.175	0.125–0.245	0.000	0.102	0.035–0.081	0.000
*n*	3193				3137	
		**Mali**				
	**Savory or sugary snacks**				**SSBs**	
	**Odds ratio**	**95% CI**	***p***	**Odds ratio**	**95% CI**	***p***
Female child	0.909	0.748–1.104	0.332	0.991	0.769–1.277	0.941
Child age (m)	1.014	1.007–1.021	0.000	1.012	1.005–1.019	0.001
Respondent completed primary education	1.455	1.112–1.904	0.007	1.379	0.975–1.950	0.069
Households in top wealth quartile	2.431	1.660–3.560	0.000	2.070	1.333–3.216	0.001
Urban area	2.887	2.024–4.116	0.000	2.504	1.609–3.897	0.000
Constant (baseline odds)	0.258	0.184–0.361	0.000	0.185	0.049–0.112	0.000
*n*	2075				2102	
		**Niger**				
	**Savory or sugary snacks**				**SSBs**	
	**Odds ratio**	**95% CI**	***p***	**Odds ratio**	**95% CI**	***p***
Female child	0.856	0.683–1.072	0.173	0.921	0.657–1.293	0.634
Child age (m)	1.028	1.021–1.034	0.000	1.034	1.018–1.050	0.000
Respondent completed primary education	2.051	1.383–3.043	0.000	2.273	1.009–5.122	0.048
Households in top wealth quartile	1.707	1.175–2.481	0.005	4.227	2.017–8.853	0.000
Urban area	2.491	1.493–4.155	0.001	2.140	0.831–5.512	0.114
Constant (baseline odds)	0.104	0.077–0.142	0.000	0.005	0.002–0.012	0.000
*n*	2651				2683	
	**Burkina Faso**					
	**Savory or sugary snacks**				**SSBs**	
	**Odds ratio**	**95% CI**	***p***	**Odds ratio**	**95% CI**	***p***
Female child	1.013	0.863–1.189	0.873	1.068	0.805–1.418	0.646
Child age (m)	1.018	1.013–1.023	0.000	1.023	1.014–1.033	0.000
Respondent completed primary education	2.076	1.738–2.481	0.000	1.920	1.238–2.979	0.004
Households in top wealth quartile	1.558	1.282–1.892	0.000	3.244	2.094–5.026	0.000
Urban area	1.938	1.577–2.381	0.000	2.838	1.713–4.702	0.000
Constant (baseline odds)	0.140	0.112–0.174	0.000	0.013	0.008–0.020	0.000
*n*	3175				3160	

Results are adjusted for sampling design.
